# Supplementary material for: Identification of Drought-Tolerance Genes in the Germination Stage of Soybean
Source: Biology (Basel). 2022 Dec 13;11(12):1812. doi: 10.3390/biology11121812 (PMC9775293; doi:10.3390/biology11121812)
Supplement: Supplementary file 1 [file biology-11-01812-s001.zip › biology-1937485-supplementary.pdf]

Figure S1 - Distribution of 200K SNP on chromosomes.

The x-axis is chromosome length, with each stripe representing a gene. Red indicate concentrated SNP. Genomes are divided into 1M sections. A is for distribution of all SNPs, B is for distribution of polymorphisms of filtered SNP.

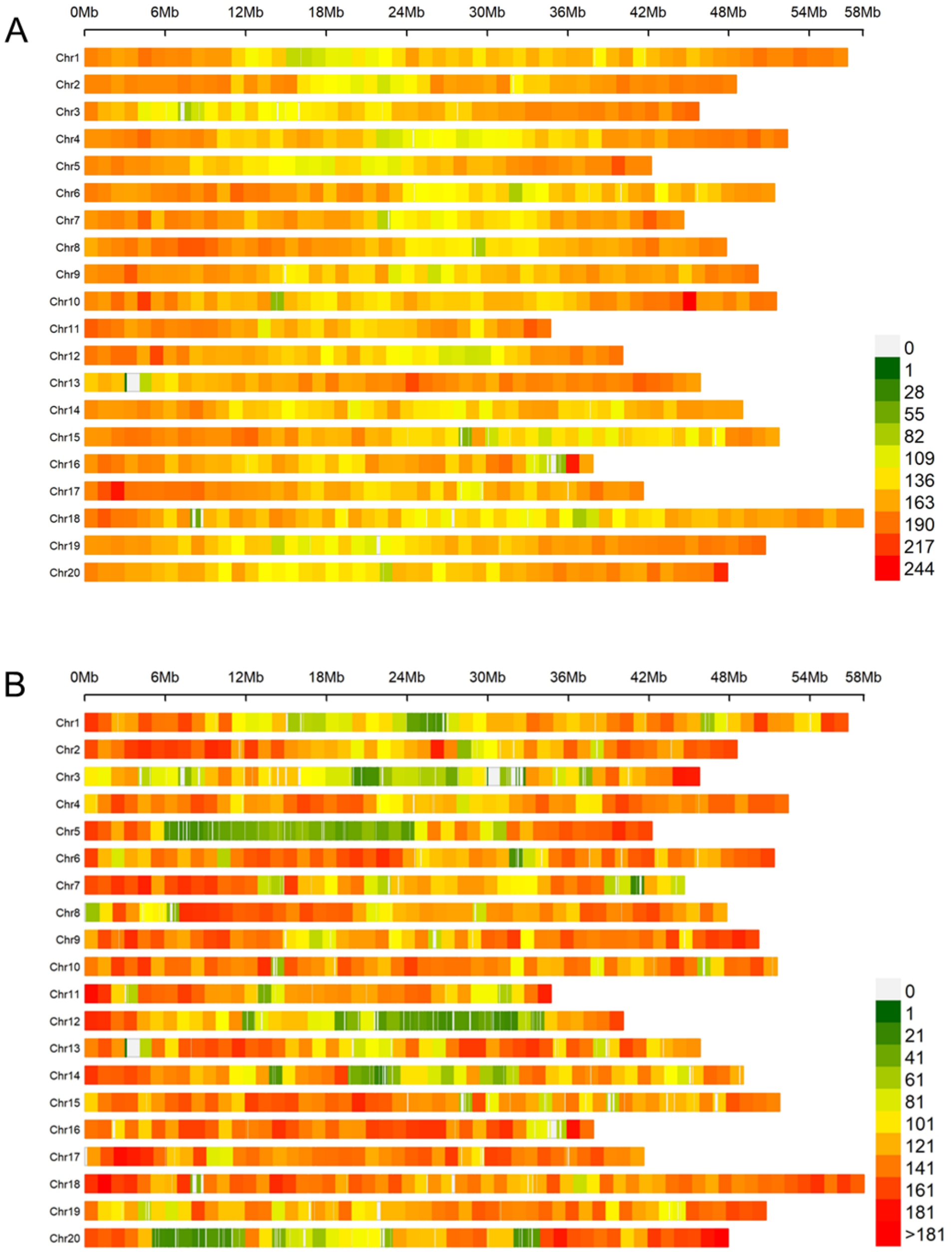

Figure S2 Identification of Gm01_48619013 Drought index loci. Local Manhattan plots and LD heatmaps (A). Locations Violinplot for drought tolerance in populations based on the genotypes for B. The middle white dot indicates the median, and the thick black bar (black box) indicates the quartile range (25% quantile and 75% quantile); GDTI: germination drought tolerant index.

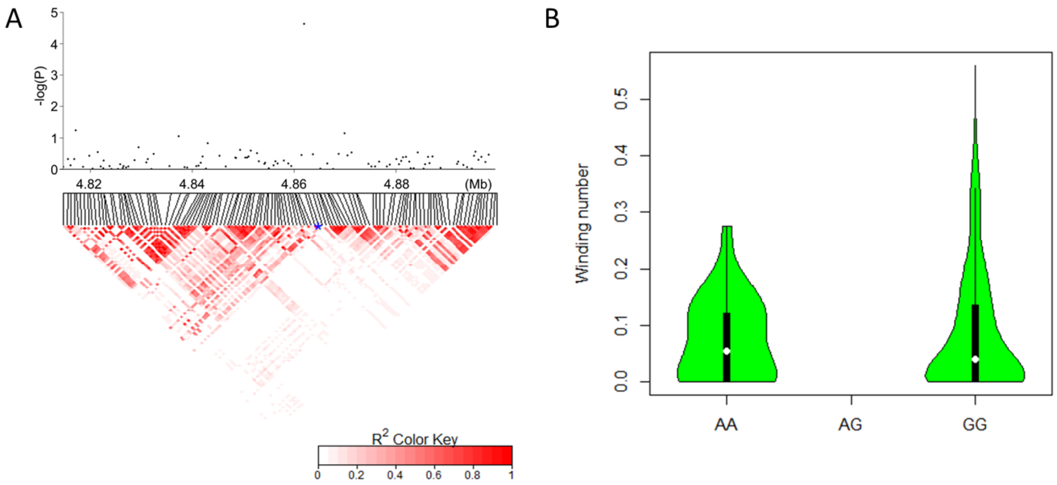

Table S1 Detailed information for the 410 soybean accessions

Test ID	Name	Geographic source
T001	BRS 132	Brazil
T002	BRS 155	Brazil
T003	Embrapa 58	Brazil
T004	С∏1271	Russia
T005	PSB471	Russia
T006	PSB543	Russia
T007	ДВ 2846	Russia
T008	ДВ 2849	Russia
T009	FUN-ZHUN/g	Russia
T010	ER-HUAN-YAN	Russia
T011	Jangbaeeg	Korea
T012	Namcheon	Korea
T013	Suwon123	Korea
T014	Bayfield	Canada
T015	CA26	Canada
T016	Rhodes	United States
T017	L60-246(Clark63)	United States
T018	L65-763	United States
T019	L67-3479	United States
T020	Harosoy	United States
T021	L65-756	United States
T022	T171	United States
T023	T219H	United States
T024	L83-544	United States
T025	L83-4387	United States
T026	L81-4590	United States
T027	Peking	United States
T028	Wilkin	United States
T029	Dunn	United States
T030	Vinton 81	United States
T031	Amsoy	United States
T032	Beeson	United States
T033	Century	United States
T034	Corsoy	United States
T035	Provar	United States
T036	Williams	United States
T037	Williams79	United States
T038	Williams82	United States
T039	Franklin	United States
T040	PI196160	United States
T041	PI157440	United States
T042	Bedford	United States
T043	DorchsoyB	United States
T044	AsgrowA1939	United States
T045	Sprite87	United States
T046	A2396	United States

T047	C1640	United States
T048	CX1038-14	United States
T049	L83-4744	United States
T050	L83-570	United States
T051	L82-1858	United States
T052	L81-4420	United States
T053	HP202	United States
T054	Amcor 89	United States
T055	Conrad	United States
T056	Newton	United States
T057	GR8836	United States
T058	Kunitz	United States
T059	Delsoy4900	United States
T060	Nile	United States
T061	Pharaoh	United States
T062	Cordell	United States
T063	Epps	United States
T064	Mack	United States
T065	Walters	United States
T066	Pickett	United States
T067	Sharkey	United States
T068	Twiggs	United States
T069	Gordon	United States
T070	Thomas	United States
T071	T308	United States
T072	T309	United States
T073	L85-1467	United States
T074	L79-842	United States
T075	L87-0482	United States
T076	Carlin	United States
T077	Accomac	United States
T078	BARC—7	United States
T079	Mercury	United States
T080	PN9394	United States
T081	Probst	United States
T082	TBD	United States
T083	9234	United States
T084	L89-2435	United States
T085	L84-2157	United States
T086	L88-8153	United States
T087	MN1301	United States
T088	Kovean	United States
T089	L72-920	United States
T090	PI 468903	United States
T091	M044	United States
T092	Tousan 83	United States
T093	Tousan kei NA5	United States
T094	Tousan kei NA75	United States
T095	Sargent	United States

T096	S99-3181	United States
T097	S01-9391	United States
T098	Osage	United States
T099	LS94-3207	United States
T100	ブローバ	Japan
T101	カニゾチ	Japan
T102	Tokachi nagaha	Japan
T103	Tsurukogane	Japan
T104	danli	Japan
T105	guandong102	Japan
T106	zhongte1	Japan
T107	Yumeyutaka	Japan
T108	AGS162	Thailand
T109	Syella	Italy
T110	Dekabig	Italy
T111	Shunyiheidou	Beijing, China
T112	Miyunlaoyelian	Beijing, China
T113	Zhongpin661	Beijing, China
T114	YZY20041515W83	Beijing, China
T115	YZY200415W90	Beijing, China
T116	AXN155	Beijing, China
T117	Zhonghuang68	Beijing, China
T118	Youhuangdou	Gansu, China
T119	Lvhuangdou	Gansu, China
T120	Tueryan	Hebei, China
T121	Baiqidawandou	Hebei, China
T122	Yangtianxiaohuangdou	Hebei, China
T123	Nanguanxiaopiqing	Hebei, China
T124	Doushidaqingdou	Hebei, China
T125	Chichenglvhuangdou	Hebei, China
T126	Datunxiaoheidou	Hebei, China
T127	Xiataizimoshidou	Hebei, China
T128	Maoyandou	Hebei, China
T129	Dongnong42A	Heilongjiang, China
T130	Dongnong42C	Heilongjiang, China
T131	Keqixiaoliheidou	Neimenggu, China
T132	Xiheheidou	Ningxia, China
T133	Loutianwaziheidou	Ningxia, China
T134	Nidinghuameidou	Ningxia, China
T135	Baipihuangdou	Shanxi, China
T136	Tianedan	Shanxi, China
T137	Tianedan	Shanxi, China
T138	Huanggandou	Shanxi, China
T139	Daheidou	Shanxi, China
T140	Chuanmanheidou	Shanxi, China
T141	Xiaheidou	Shanxi, China
T142	Hongdadou	Shanxi, China
T143	Xiaohuangdou	Shanxi, China
T144	Gingkeyuandou	Shanxi, China

T145	Huangdou2	Shanxi, China
T146	Xiaohuangdou	Shanxi, China
T147	Yuxuan13hao	Shanxi, China
T148	Bailudou	Shanxi, China
T149	Liushiribaidou	Shanxi, China
T150	Xiaobaidou2	Shanxi, China
T151	Xiaoheidou	Shanxi, China
T152	Dongshan69	Shanxi, China
T153	Lvpihuangdou	Shanxi, China
T154	Tiefeng31	Shanxi, China
T155	Zaoshuhuangdou	Shanxi, China
T156	Xiaoheidou	Shanxi, China
T157	Xiaoheidou	Shanxi, China
T158	Laoheidou	Shanxi, China
T159	Yanqihuangdou	Xinjiang, China
T160	Changjihuangdou1	Xinjiang, China
T161	Dongnong4	Heilongjiang, China
T162	Fengshou1	Heilongjiang, China
T163	Heihe1hao	Heilongjiang, China
T164	Mufeng1	Heilongjiang, China
T165	Suinong1hao	Heilongjiang, China
T166	Jingshanpu	Heilongjiang, China
T167	Tujiazi	Heilongjiang, China
T168	Baimaoshuang	Heilongjiang, China
T169	Liushitianhuanjia	Heilongjiang, China
T170	Huananxiaojindou	Heilongjiang, China
T171	Qingdou	Heilongjiang, China
T172	Lvrangheidou	Heilongjiang, China
T173	Qinganheidou	Heilongjiang, China
T174	Fangzhengmoshidou	Heilongjiang, China
T175	Nenfeng11hao	Heilongjiang, China
T176	Hefeng24hao	Heilongjiang, China
T177	Hefeng25hao	Heilongjiang, China
T178	Dongnong36hao	Heilongjiang, China
T179	Heihexiaohuangdou	Heilongjiang, China
T180	Longquandadou(heqi)	Heilongjiang, China
T181	Xiaolimoshidou	Heilongjiang, China
T182	Suinong14hao	Heilongjiang, China
T183	Hedou2(MN413)	Heilongjiang, China
T184	Heihe38	Heilongjiang, China
T185	Hefeng52	Heilongjiang, China
T186	Heinong47	Heilongjiang, China
T187	HLT2	Heilongjiang, China
T188	Ha123510	Heilongjiang, China
T189	Jilin3	Jilin, China
T190	Xiaojinhuang1	Jilin, China
T191	Fengdihuang	Jilin, China
T192	Jinyuan1	Jilin, China
T193	Ha1	Jilin, China

T194	Huichundou	Jilin, China
T195	Jiaohezihua1	Jilin, China
T196	Lanqi	Jilin, China
T197	Changchunmancangjin	Jilin, China
T198	Niumaohuang	Jilin, China
T199	Baodigao	Jilin, China
T200	Chasedou	Jilin, China
T201	Heimoshidou	Jilin, China
T202	Heimodou	Jilin, China
T203	Zihua2hao	Jilin, China
T204	Fuyuduludou	Jilin, China
T205	Jiutaibaodigao	Jilin, China
T206	Huaidebaihuadali	Jilin, China
T207	Helongyoutai	Jilin, China
T208	Tonghuapingdingxiang	Jilin, China
T209	Baichengmoshidou	Jilin, China
T210	Jinshanchamoshidou	Jilin, China
T211	Jilinchalihua	Jilin, China
T212	Huangdali	Jilin, China
T213	Hefeng37hao	Jilin, China
T214	Dongsheng1	Jilin, China
T215	Jilin30	Jilin, China
T216	Jiyu67	Jilin, China
T217	Jilinxiaolidou	Jilin, China
T218	Jiyu86	Jilin, China
T219	Jiyu109	Jilin, China
T220	Tiefeng18	Liaoning, China
T221	Jindou33	Liaoning, China
T222	Jinzhou41	Liaoning, China
T223	Dabaimei	Liaoning, China
T224	Tianedan	Liaoning, China
T225	Daheiqi	Liaoning, China
T226	Heiqi	Liaoning, China
T227	Dadou2	Liaoning, China
T228	Tiejiajinping	Liaoning, China
T229	Huangqi	Liaoning, China
T230	Xiaobaiqi	Liaoning, China
T231	Xiaohuangdou	Liaoning, China
T232	Niumaohuang	Liaoning, China
T233	Qingpipingdingxiang	Liaoning, China
T234	Baitiejia	Liaoning, China
T235	Baiheidou	Liaoning, China
T236	Daliheidou	Liaoning, China
T237	Liushitianhuancang	Liaoning, China
T238	Yushidou	Liaoning, China
T239	Jiyu72	Liaoning, China
T240	Liaodou11	Liaoning, China
T241	Liaodou16	Liaoning, China
T242	Dongnong50	Liaoning, China

T243	Tiefeng29	Liaoning, China
T244	Liaodou32	Liaoning, China
T245	Liao08012	Liaoning, China
T246	Liao08Q104	Liaoning, China
T247	Liao08024	Liaoning, China
T248	Liao10Q015	Liaoning, China
T249	Chi382	Neimenggu, China
T250	Jindou36	Neimenggu, China
T251	Suiningpingdinghuang	Jiangsu, China
T252	Pixianhongmaoyou	Jiangsu, China
T253	Pixiandazihuacao	Jiangsu, China
T254	Pixiansilicao	Jiangsu, China
T255	Huaiyangchundou	Jiangsu, China
T256	Muyangchunheidoubing	Jiangsu, China
T257	Pudou206	Jiangsu, China
T258	Hualvhuangdou	Gansu, China
T259	Diliuhuangdou2	Hebei, China
T260	Sijiaoqihuangdou	Hebei, China
T261	Bendidahuangdou	Hebei, China
T262	Heidou	Hebei, China
T263	Huaheihu	Hebei, China
T264	Jidou7hao	Hebei, China
T265	Qingdou	Hebei, China
T266	Miyangxiaozihuang	Henan, China
T267	Xichuanjiwohuang	Henan, China
T268	Miyangniumaohuang	Henan, China
T269	Zhechengxiaohongdou	Henan, China
T270	Boaihongpizaojiaozi	Henan, China
T271	Xinyangyangyandou	Henan, China
T272	Zheng8516	Henan, China
T273	Zheng84240B1	Henan, China
T274	Shanning7	Henan, China
T275	Pixianlayanghuang	Jiangsu, China
T276	Tongshanqingdadou	Jiangsu, China
T277	Guanyunhaibaihua	Jiangsu, China
T278	Sidou2hao	Jiangsu, China
T279	Shengli3hao	Shandong, China
T280	Siliyuan	Shandong, China
T281	Pingdinghuangdou	Shandong, China
T282	Dabaipi	Shandong, China
T283	Dahuangdou	Shandong, China
T284	Datianedan	Shandong, China
T285	Xiaomidou	Shandong, China
T286	Lvcaodou	Shandong, China
T287	Douliheidou	Shandong, China
T288	Pingdinghei	Shandong, China
T289	Chadou	Shandong, China
T290	Maodou	Shandong, China
T291	Qisiwa	Shandong, China

T292	Gaozuoxuan1hao	Shandong, China
T293	Jilin36	Shandong, China
T294	Mengdou14	Shandong, China
T295	Niumaohuang	Shanxi, China
T296	Huichaxiaohuangdou	Shanxi, China
T297	Niupihuangdou	Shanxi, China
T298	Laoshupi	Shanxi, China
T299	Jianghuangdou	Shanxi, China
T300	Baomuji	Shanxi, China
T301	ZDD04918	Anhui, China
T302	ZDD04959	Anhui, China
T303	WeiJ127	Anhui, China
T304	Jindou21	Anhui, China
T305	Huaidou4	Anhui, China
T306	Doushanbaimadou	Fujian, China
T307	Dalihuang	Fujian, China
T308	Daqingren	Fujian, China
T309	Xiamentengzidou	Fujian, China
T310	Tonganzihongdou	Fujian, China
T311	Pudou451	Fujian, China
T312	Quanbian11	Fujian, China
T313	Zhaoanqiudadou	Fujian, China
T314	Shaxianqingdou	Fujian, China
T315	Shaxianwudou	Fujian, China
T316	Baiqiu1hao	Fujian, China
T317	Dabaimaodou	Guangdong, China
T318	Longchuanhuangniumao	Guangdong, China
T319	Lianjiangpohuangdou	Guangdong, China
T320	Qingyuandaqingdou	Guangdong, China
T321	Yingdehedou	Guangdong, China
T322	Dahuangdou2	Guangdong, China
T323	Madaiqingdou2	Guangdong, China
T324	Doupingqingdou	Guangdong, China
T325	Madaiheidou3	Guangdong, China
T326	Baizhidou	Guangxi, China
T327	Dawudou	Guangxi, China
T328	Mashanrenfenghuangdou	Guangxi, China
T329	Daimaodou	Guizhou, China
T330	Xihuangdou8	Guizhou, China
T331	Xihuangdou9	Guizhou, China
T332	Doujizaodou2	Guizhou, China
T333	Zaohuangdou	Guizhou, China
T334	Dahuangdou1	Guizhou, China
T335	Zaojiaodou	Guizhou, China
T336	Zadou6	Guizhou, China
T337	Qiyuehuang1	Guizhou, China
T338	Heikewudou	Hainan, China
T339	Jinghuang35yi	Hubei, China
T340	Daimidou	Hubei, China

T341	Zhongdou24	Hubei, China
T342	8216	Hubei, China
T343	Chihuangdou2	Hubei, China
T344	Shuguanghuangdou	Hubei, China
T345	Chahuangdaidou1	Hubei, China
T346	Shanzibaihuangdou	Hubei, China
T347	Chihuangdou1	Hubei, China
T348	Huameidou	Hubei, China
T349	Xiaokehuangdou	Hubei, China
T350	Honghuliuyuebao	Hubei, China
T351	Nidou	Hubei, China
T352	8470	Hubei, China
T353	Huangmaodou	Hunan, China
T354	Hongzhudou	Hunan, China
T355	Changshanidou	Hunan, China
T356	Aishengnidou1	Hunan, China
T357	Yizhangliuyuehuang	Hunan, China
T358	Wujiangwuyueniumaohuang	Jiangsu, China
T359	Yizhengdalihuangdou	Jiangsu, China
T360	Taixingheidou	Jiangsu, China
T361	Taixingaijiaohong	Jiangsu, China
T362	77-391-1	Jiangsu, China
T363	Shaxindou	Jiangxi, China
T364	Ruijinqingpidou	Jiangxi, China
T365	Dahuangzhu	Jiangxi, China
T366	Xinyudaliqing	Jiangxi, China
T367	Shangraobayuebai	Jiangxi, China
T368	Yantianqingpidou	Jiangxi, China
T369	Hengfengwudou	Jiangxi, China
T370	Wuyuehuang	Jiangxi, China
T371	Duchangwudou	Jiangxi, China
T372	Fengchengzaowudou	Jiangxi, China
T373	Dahuadou	Sichuan, China
T374	Wuyanwo	Sichuan, China
T375	Shiyuehuang	Sichuan, China
T376	Zengjialvhuangdou	Sichuan, China
T377	Jiangehualinjiwodou	Sichuan, China
T378	Qionglaihuangmaozi	Sichuan, China
T379	Qionglaiyoujiangheidou	Sichuan, China
T380	Hanyuanbalixiaoheidou	Sichuan, China
T381	Douhuangdou1	Sichuan, China
T382	Liuyuebao2	Sichuan, China
T383	Zaohuangdou4	Sichuan, China
T384	Baimaozaodouzi	Sichuan, China
T385	Touxinlv	Sichuan, China
T386	Lvdouzi	Sichuan, China
T387	Lvlanzi	Sichuan, China
T388	Xiaobaimao	Sichuan, China
T389	Bazhongtiankandou2	Sichuan, China

T390	Quxianbayuehuang	Sichuan, China
T391	Pixianxiaohuangdou	Sichuan, China
T392	Zizhongliuyuezao	Sichuan, China
T393	Jianweiquanshuidou	Sichuan, China
T394	Changshoushiyuehuang	Sichuan, China
T395	Suiningfengtaijiangsedou	Sichuan, China
T396	Shifangluosidou	Sichuan, China
T397	8307-8-1	Sichuan, China
T398	Gongdou7hao	Sichuan, China
T399	Liuyuehuang	Sichuan, China
T400	Pengshanhuangkezi3	Sichuan, China
T401	Xicangdadou12	Xicang, China
T402	Xuanza	Yunnan, China
T403	Huangdou	Yunnan, China
T404	Yangyandou	Yunnan, China
T405	Songzidou	Yunnan, China
T406	Malanzaochadou	Yunnan, China
T407	Zaoshumaopengqing	Zhejiang, China
T408	Cudou	Zhejiang, China
T409	Fudou9765	Zhejiang, China
T410	Quxian3	Zhejiang, China

Table S2 Detailed information for the six soybean accessions used for PEG screening

Test ID	Name	Geographic source
D001	Muyangchunheidoubing	Jiangsu, China
D002	Xiaomidou	Shandong, China
D003	Douliheidou	Shandong, China
D004	Jindou21	Anhui, China
D005	Qisiwa	Shandong, China
D006	Sidou2hao	Jiangsu, China

Table S3 Descriptive statistics of four germination-related traits under 0% PEG (C) and 20% PEG (D) conditions for the 410 soybean accessions

Traits	Treat	Range	Mean	SD	CV
GR	D	0.00~100.00	15.07	18.69	124.01
	C	60.00~100.00	96.92	5.95	6.14
GE	D	0.00~96.67	13.58	17.37	127.92
	C	55.00~100.00	96.33	6.78	7.04
GDI	D	0.00~135.83	17.49	23.40	133.80
	C	82.50~250.00	189.20	35.12	18.56
GI	D	0.00~4.69	0.68	0.86	127.79
	C	2.61~14.92	7.50	1.90	25.36

GR, germination rate; GE, germination energy; GDI, germination drought index; GI, germination index; *SD* standard deviation; *CV* coefficient of variation.

Table S4 Genetic parameters revealed by the analysis of 117,811 polymorphic SNP markers in the 410 soybean accessions

	Minimum	Maximum	Mean
Minor allele frequency	0	0.5030	0.2228
Gene diversity	0	0.5061	0.3043
Heterozygosity	0	0.4070	0.0237
PIC	0	0.3843	0.2458

Table S5 SNPs positioned near genes and functional annotated information for these genes

Marker	Chr.	Position	Site	Gene	Homologous gene in Arabidopsis	Functional annotation
Gm01_35877607	1	35877607	Intergenic region	Glyma.01g106000; Glyma.01g106100	AT3G09270	Glutathione S-transferase U1-related
Gm01_38948188	1	38948188	Intergenic region	Glyma.01g113500; Glyma.01g113600	AT2G24670	Domain of unknown function
Gm01_47042336	1	47042336	Intergenic region	Glyma.01g141000; Glyma.01g141100	AT5G12060	Genomic DNA, chromosome 3, P1 clone: MDJ14-related
Gm01_48619013	1	48619013	nonsynonymous	Glyma.01g149300	AT1G31850	Methyltransferase PMT21-related
Gm02_6357585	2	6357585	synonymous	Glyma.02g072600	AT3G03860	5'-adenylylsulfate reductase-like 5-related
Gm03_39037	3	39037	Intergenic region	Glyma.03g000200; Glyma.03G000300	AT2G31820	Ankyrin repeats-containing protein
Gm04_4484515	4	4484515	Intergenic region	Glyma.04g055500; Glyma.04g055600	AT1G76880	NA
Gm04_50945875	4	50945875	nonsynonymous	Glyma.04g241400; Glyma.03G000300	AT1G21460	Bidirectional sugar transporter sweet1
Gm05_38540838	5	38540838	Intergenic region	Glyma.05g201700; Glyma.05G201800	AT5G50915	Transcription factor BLLH137
Gm06_9791913	6	9791913	nonsynonymous	Glyma.06g120400	AT1G55200	Interleukin-1 receptor-associated kinase 1 (IRAK1)
Gm07_24735482	7	24735482	Intergenic region	Glyma.07G165100; Glyma.07g165200	AT2G43630	Glycine-rich protein
Gm08_1438457	8	1438457	intronic	Glyma.08g017800	AT1G63940	Monodehydroascorbate reductase, chloroplastic
Gm08_4052111	8	4052111	nonsynonymous	Glyma.08g052100	AT3G18050	Genomic DNA, Chromosome 3, P1 Clone: MRC8
Gm08_7972856	8	7972856	synonymous	Glyma.08g103900	AT1G67980	Flavonoid 3',5'-methyltransferase
Gm09_11414508	9	11414508	Intergenic region	Glyma.09g087500; Glyma.09g087600	AT1G09040	Atrophin-related // subfamily not named
Gm09_18023730	9	18023730	Intergenic region	Glyma.09g099300; Glyma.09g099400	AT3G42170	Finger-related // subfamily not named
Gm11_30280479	11	30280479	intronic	Glyma.11g210400	AT2G18950	homogentisate phytyltransferase / homogentisate geranylgeranyltransferase
Gm13_35517964	13	35517964	synonymous	Glyma.13g246400	NA	NA
Gm14_46603856	14	46603856	Intergenic region	Glyma.14g200900; Glyma.14G201100	AT4G35160	O-methyltransferase// subfamily not named
Gm15_11950665	15	11950665	UTR3	Glyma.15g145200	AT4G16110	Response regulator of two-component system // subfamily not named
Gm15_47429024	15	47429024	Intergenic region	Glyma.15g248700; Glyma.15G248800	AT2G01050	Domain of unknown function (DUF4283)
Gm19_49449499	19	49449499	UTR3	Glyma.19g248400	AT3G04490	Exportin-4
Gm20_4618170	20	4618170	Intergenic region	Glyma.20g033800; Glyma.20G033900	AT5G15290	Casparian strip membrane protein 5

Gm20_13921498	20	13921498	Intergenic region	Glyma.20g056300 ; Glyma.20G056400	NA	gag-polypeptide of LTR copia-type (UBN2)
Gm20_34956219	20	34956219	Intergenic region	Glyma.20G106800 ; Glyma.20g106900	AT1G34360	Translation initiation factor IF-3 // subfamily not named
Gm20_36902659	20	36902659	UTR3	Glyma.20g126800	AT4G35220	Arylformamidase / Kynurenine formamidase
